# Four Simple Biomimetic Mineralization Methods to Improve the Thermostability and Immunogenicity of Virus-like Particles as a Vaccine against Foot-and-Mouth Disease

**DOI:** 10.3390/vaccines9080891

**Published:** 2021-08-12

**Authors:** Mengnan Guo, Jiajun Li, Zhidong Teng, Mei Ren, Hu Dong, Yun Zhang, Jiaxi Ru, Ping Du, Shiqi Sun, Huichen Guo

**Affiliations:** 1 State Key Laboratory of Veterinary Etiological Biology, National Foot-and-Mouth Disease Reference Laboratory, Lanzhou Veterinary Research Institute, Chinese Academy of Agricultural Sciences, Lanzhou 730030, China; guomengnan2021@163.com (M.G.); jiajunlii@163.com (J.L.); tengzhidong163@163.com (Z.T.); Mei.Ren@student.uliege.be (M.R.); donghu.0608@163.com (H.D.); zhangyun03@caas.cn (Y.Z.); rujiaxi@caas.cn (J.R.); westernboydp555@163.com (P.D.); sunshiqi@caas.cn (S.S.); 2 College of Animal Science, Yangtze University, Jingzhou 434025, China; 3 Yunnan Tropical and Subtropical Animal Virus Diseases Laboratory, Yunnan Animal Science and Veterinary Institute, Kunming 650000, China

**Keywords:** foot-and-mouth disease, virus-like particles, biomineralization, thermal stability, biomimetic

## Abstract

The need for a cold chain system during storage and transport substantially increases the cost of vaccines. Virus-like particles (VLPs) are among the best countermeasures against foot and mouth disease virus (FMDV). However, VLPs are composed of pure proteins, and thus, are susceptible to heat. To address this problem, four simple biomimetic mineralization methods with the use of calcium phosphate were developed to improve heat tolerance via biomineralization. The results showed that biomineralization can significantly improve the heat resistance of VLPs. The biomineralized VLPs can be stored at low as 25 °C for eight days, and 37 °C for four days. Animal experiments showed that biomineralization had no effect on the immunogenicity of VLPs or the expression of specific antibodies (Abs) and neutralizing Abs. Even after heat treatment at 37 °C for four days, the biomineralized VLPs remained immunogenic and produced highly specific and neutralizing Abs with a high rate of protection. These results suggest that these biomineralization approaches can promote the thermal stability of VLPs against and significantly reduce dependence on cold storage and delivery systems.

## 1. Introduction

Foot and mouth disease (FMD), caused by the foot-and-mouth disease virus (FMDV), is a highly infectious disease of even-hoofed animals such as cattle, pigs, sheep, goats, and deer, and rapidly spreads, either directly or indirectly, among susceptible animals [1,2]. FMDV exists as seven immunologically distinct serotypes, which are further classified into multiple subtypes [3]. Outbreaks of FMD cause massive economic losses to the animal husbandry industry worldwide. Many countries including most in the European Union have implemented strict legislation and control strategies to eradicate FMD [4]. Nonetheless, outbreaks of FMD remain a continual threat. Notably, more than 3.5 million animals were culled due to an outbreak of FMD in South Korea in November 2010 [5]. Although there is currently no consensus on a treatment regimen against the method to treat FMD, the use of inactivated vaccines is the most effective control measure to date [6]. Conventional vaccines are highly effective against FMD, but can cause problems, especially the introduction of more virulent strains [7].

Virus-like particles (VLPs), as novel vaccine candidates, are supra-molecular multi-protein structures that mimic the organization and conformation of the parental virus, but lack the viral genome, and thus are potentially safer and cheaper alternatives [8,9,10]. Like inactivated viruses, VLPs can activate high levels of neutralizing antibodies (Abs). The rigid and repetitive surface structures of VLPs can induce strong B-cell responses in the absence of adjuvants by efficiently cross-linking specific surface receptors on B cells and can produce single-domain Abs such as M8 and M170 with better neutralizing activities [11], thus rendering these particles as highly immunogenic vaccine templates [12,13]. The development of VLP-based vaccines has been ongoing for more than 30 years. Thus far, 110 proteins produced by 35 viruses have been assembled into VLPs [14]. At present, six VLP-based vaccines including Gardasil^®^, Cervarix^®^, Hecolin^®^, and PorcilisPCV^®^ have been confirmed as both safe and effective for clinical use.

VLPs naturally occur or are synthesized to express viral structural proteins that can self-assemble into virus-like structures [15]. VLP-based vaccines such as human papilloma virus [16] and hepatitis B virus [17] have already been applied clinically. VLPs can be produced by multi-cell culture systems of yeast, *Escherichia coli* (*E. coli*), baculoviruses, and mammalian cells [18]. As a predominant protein expression host, *E. coli* has many advantages such as low cost, high expression, ability to easily scale-up the culture, and short turnaround time [19]. Xiamen Innovax Biotech (Xiamen, China) has used *E. coli* to produce a vaccine against human papilloma virus based on L1 VLP at a significantly lower cost than that of the current eukaryotic systems [20]. Our group previously reported the successful development of VLPs FMDV [21]. VLPs are proteins similar to the inactivated vaccines against FMDV, but with no inner nucleotides. Since most biological products are highly heat-sensitive, a cold chain system is necessary for the global procurement and distribution of vaccines [22].

A cold chain system guarantees the thermal stability of vaccines, but remains a major economic and logistical obstacle to global vaccine distribution, and can increase the cost by up to 80% [23,24]. Furthermore, a global network of cold rooms, freezers, refrigerators, cold boxes, and carriers is needed to successfully execute a global vaccination program, but can damage the quality of the vaccine. For example, a break in a cold chain system during storage and transportation can result in losses of up to 50% of the vaccine suitable for administration [25]. Various methods to improve the thermal stability of vaccines and many stable formulations have been developed with the use of trehalose, MgCl_2_, and non-reducing sugars. However, although these technological advances have improved the stability of vaccines, interactions between the capsid and nucleic acids are increased [25]. In addition, these methods involve complex processing procedures. Biomineralization is the simplest and most effective technique to improve the thermal stability of vaccines.

Biomineralization is a process in which biological materials precipitate mineral materials, as occurs with the formation and maintenance of teeth, bones, and eggshells [26]. In nature, biomineralization is employed by organisms to produce various biological minerals to ensure survival in response to harsh environments. For example, calcium phosphate (CaP) in eggshells allows for long-term storage of eggs under various environmental conditions [27]. In addition, biosilicification allows for the survival of the archaeal bacteriophages in response to high-temperature conditions [28].

Acidic amino acids on the protein surface can serve as binding sites for factors that regulate biomineralization [29,30]. Hence, we hypothesized that the biomineralization process could be exploited to artificially confer CaP as a thermal protective shell to encapsulate VLPs. Therefore, four simple biomimetic mineralization methods were developed to generate CaP shells to protect heat-susceptible VLPs against FMD. The biomineralized VLPs exhibited improved thermal stability and were still effective after more than four days of storage at 37 °C. The CaP shells largely prevent direct contact between the interior of the VLPs and the outside environment. CaP shells were fabricated to provide a physical barrier and prevent structural changes to the VLPs. The CaP shells serve as a physical barrier to prevent fundamental changes to VLPs.

In this study, we discuss four original biomineralization methods that significantly improved the heat resistance of VLPs against FMD (FMD-VLPs). Experiments with the use of guinea pigs showed that the biomineralized VLPs produced higher Ab titers and offered better protection against FMD. These findings can help to significantly improve the heat resistance of vaccines and reduce the need for cold-chain storage and transportation.

## 2. Materials and Methods

### 2.1. Preparation of FMD-VLPs

The *E.coli* system employed for the expression, purification, and assembly of FMD-VLPs is described in our previous report [21]. Under denaturing conditions, protein expression levels were assessed by sodium dodecyl sulfate−10% polyacrylamide gel electrophoresis and the concentrations of FMD-VLPs were determined using the Pierce™ BCA Protein Assay Kit (Thermo Fisher Scientific, Waltham, MA, USA) in accordance with the manufacturer′s instructions. The VLPs (1.5 mL) were purified through a sucrose gradient (15−45%, *w*/*w*) and ultracentrifuged at 4 °C at 38,000× *g* for 4 h with an Optima L-100 XP Ultracentrifuge (Beckman Coulter, Fullerton, CA, USA) with a SW 41 Ti Swinging-Bucket Rotor (Beckman Coulter). The size and morphological characteristics of the VLPs were observed using dynamic light scattering (DLS; ZEN3600, Malvern, PA, USA) and a transmission electron microscope (TEM; HT7700; Hitachi Corporation, Tokyo, Japan).

### 2.2. Biomineralization of FMD-VLPs

Four biomineralization methods were used to prepare the VLPs-CaP complexes, as follows:

Method 1: 200 μg of VLPs were mixed with 1400 μL (1.5 mM) of (NH_4_) _2_HPO_4_ and 200 μL (17.5 mM) of Ca (NO_3_)_2_ and stirred. The resulting biomineralization product was labeled as VLPs-CaP-1.

Method 2: 200 μg of VLPs were mixed with 1600 μL (1.25 mM) of (NH_4_)_2_HPO_4_ and 200 μL (16.7 mM) of Ca (NO_3_)_2_ and stirred. The resulting mineralized product was labeled as VLPs-CaP-2.

Method 3: 200 μg of VLPs were mixed with 1400 μL (1.5 mM) of Na_2_HPO_4_ and 200 μL (16.7 mM) of CaCl_2_ and stirred. The resulting mineralized product was labeled as VLPs-CaP-3.

Method 4: 200 μg of VLPs was mixed with 1600 μL (1.25 mM) of Na_2_HPO_4_ and 200 μL (16.7 mM) of CaCl_2_ and stirred. The resulting mineralized product was labeled as VLPs-CaP-4.

The biomineralized VLPs were separated by centrifugation at 13,000 rpm× *g* for 10 min. The biomineralization efficiency was determined by calculating the amount of VLPs in the supernatant relative to the sample.

Biomineralization efficacy =

(1) $$\left(1-\frac{\mathrm{Amount}\;\mathrm{of}\;\mathrm{VLPs}\;\mathrm{in}\;\mathrm{supernatant}}{\mathrm{Total}\;\mathrm{amount}\;\mathrm{of}\;\mathrm{VLPs}\;\mathrm{in}\;\mathrm{loading}\;\mathrm{samples}}\right)\times 100\%$$

### 2.3. Characterization and Measurements

The hydrodynamic diameters and zeta potentials were measured with a DLS and a Zetasizer Nano ZS instrument, respectively. TEM observations were performed using an HT7700 instrument. For TEM measurements, the samples were dropped onto carbon-coated copper TEM grids (400 mesh, Agar Scientific, Stansted, England) and dried at room temperature before observation. Scanning electron microscopy (SEM) and energy-dispersive X-ray (EDX) spectroscopy were conducted using a JSM-6701F instrument (JEOL, Tokyo, Japan). Selected area electron diffraction analysis was performed using a TF20 instrument (FEI, Hillsboro, OR, USA). Fourier transform infrared (FTIR) spectroscopy was performed using a Nicolet Nexus 670 instrument (Thermo Fisher Scientific).

### 2.4. Cell Viability (and In Vitro Cytotoxicity of the VLPs-CaP Composites)

The cytotoxicity of the VLPs-CaP composites was assessed using the MTS (3-(4,5-dimethylthiazol-2-yl)-5-(3-carboxymethoxyphenyl)-2-(4-sulfophenyl)-2H-tetrazolium) Assay (Cell Titer 96^®^ AQueous One Solution Cell Proliferation Assay; Promega Corporation, Madison, WI, USA). Baby hamster kidney fibroblasts (BHK-21) cells and immortalized bone-marrow-derived macrophages (iBMM) were incubated in DMEM culture medium consisting of 10% (*v*/*v*) FBS supplemented with 100 U/mL penicillin and 100 μg/mL streptomycin. The cells were seeded into 96-well culture plates (1 × 10^4^ cells per well). The plates were incubated under 5% CO_2_ at 37 °C. After 24 h, FMD VLPs, VLPs-CaP-1, VLPs-CaP-2, VLPs-CaP-3, and VLPs-CaP-4 with different concentrations (10, 30, 50, 70, and 100 μg/mL) were added into cells and incubated at 37 °C under 5% CO_2_ for 24 h and 48 h. According to the MTS manual, we used the MTS method to determine the viability and cytotoxicity of VLPs and VLPs-CaP.

### 2.5. Cellular Uptake

BHK-21 cells and iBMM were seeded into the wells of 6-well culture plates (2 × 10^6^ cells/well) and incubated under an atmosphere of 5% CO_2_ at 37 °C for 48 h. Afterward, 5 μg of the FMD VLPs, VLPs-CaP-1, VLPs-CaP-2, VLPs-CaP-3, and VLPs-CaP-4, respectively, were added to the wells and the cells were incubated under an atmosphere of 5% CO_2_ at 37 °C for 2, 4, and 6 h. After washing with phosphate-buffered saline (PBS), the cells were collected for western blot analysis of FMD-VLPs. The results were analyzed with ImageJ software (Fiji, ImageJ, ver 1.8.0).

### 2.6. Thermal Stability Analysis

The FMD-VLPs, VLPs-CaP-1, VLPs-CaP-2, VLPs-CaP-3, and VLPs-CaP-4 were respectively incubated at either 24 °C or 37 °C and samples were collected every day. The dot blot method was used to quantify FMD-VLPs. Briefly, 7 μL of the FMD-VLPs were added to a nitrocellulose filter membrane for 1 h at 37 °C sealed with 5% skimmed milk for 1 h and probed with primary Abs against the FMD-VLPs for 1 h, followed by anti-mouse immunoglobulin G secondary Abs (Sigma-Aldrich Corporation, St. Louis, MO, USA) for 1 h. Afterward, the blots were visualized using a chemiluminescent imaging system (Tanon 4600; Tanon Science & Technology Co. Ltd., Shanghai, China).

VLPs-CaP-1, VLPs-CaP-2, VLPs-CaP-3, and VLPs-CaP-4 were stored at 25 °C and 37 °C, respectively, and samples were collected periodically. The morphological characteristics of the samples were observed by TEM (Hitachi High-Technologies Corporation, Tokyo, Japan).

### 2.7. Animal Experiments

Animal experiments were performed to assess the immune effect of biomineralized VLPs with the use of 48 male Hartley guinea pigs (average body weight, 200 g) obtained from the Lanzhou Veterinary Research Institute (Lanzhou, China), which were randomly assigned to a control (PBS) group or one of five treatment groups. The guinea pigs were injected with 1 mL of PBS, FMD VLPs, VLPs-CaP-1, VLPs-CaP-2, VLPs-CaP-3, or VLPs-CaP-4 in the tibialis cranial muscles of both rear legs. Each of the vaccine preparations contained 20 μg of the VLPs. Whole blood samples were collected from the hearts of the guinea pigs on immunization days 7, 14, 21, and 28, and centrifuged to obtain serum samples. Twenty-eight, with four guinea pigs in each group, were randomly selected and challenged with 200 μL FMDV type O strain O/BY/CHA/2010 (GenBank accession no. JN998085.1) at a 100 guinea pig intraperitoneal 50% infectious dose via inoculation of the left heel pads. Guinea pigs were immunized with FMDV in a bio-safety level 3 animal facility at the State Key Laboratory of Veterinary Etiological Biology (Lanzhou, China).

Further animal experiments were conducted to assess the thermal stability of the biomineralized VLPs with the use of 48 male Hartley guinea pigs (average body weight, 200 g) obtained from the Lanzhou Veterinary Research Institute that were randomly assigned to a control or one of five treatment groups. The animals were injected with PBS, FMD-VLPs (stored at 37 °C for two days), VLPs-CaP-1 (stored at 37 °C for four days), VLPs-CaP-2 (stored at 37 °C for four days), VLPs-CaP-3 (stored at 37 °C for four days), or VLPs-CaP-4 (stored at 37 °C for four days). Before storage, the vaccine preparations contained 20 μg of the VLPs. On post-vaccination day 28, six guinea pigs from each group were randomly selected for the challenge experiment.

### 2.8. Serum Neutralization

The titers of the neutralizing Abs induced by the different vaccine preparations were measured using a microtiter neutralization assay with BHK-21 cells. Serial dilutions of serum (50 μL) were incubated with an equal volume of FMDV type O strain O/BY/CHA/2010 at a 100 guinea pig intraperitoneal 50% infectious dose for 1 h at 37 °C and four replications were prepared for each dilution. The mixture was then added to 50 μL of cells plated at a density of 2 × 10^4^ per well. The cells were then incubated for three days. Afterward, the cytopathic effect was observed under a microscope. The Reed–Muench method was used to calculate the serum dilution of the positive result. The titer of the neutralization Abs was calculated as log 10 of the reciprocal of the highest dilution causing 50% neutralization.

### 2.9. Statistical Analysis

Statistical analyses were conducted using one-way analysis of variance (ANOVA) with GraphPad Prism 8.0.1 software (GraphPad Software, Inc., San Diego, CA, USA). A probability (*p*) value of <0.05 was considered statistically significant and <0.01 as highly significant.

## 3. Results

### 3.1. Preparation and Characterization of the VLPs-CaP Complexes

In order to obtain biomineralized FMD-VLPs, the assembly of the FMD-VLPs was confirmed with DLS and TEM, and the particle size and morphology were characterized. The FMD-VLPs were spherical particles with diameters of about 25–40 nm (Supplementary Figure S1).

To induce biomineralization, the VLPs were incubated in phosphate and calcium mineralization solutions. Then, the mineralized VLPs were centrifuged at 13,000 rpm× *g* for 10 min, while the unmineralized VLPs were centrifuged through a sucrose gradient at 38,000 rpm× *g* for 3 h because biomineralization had increased the mass of the VLPs. Equal amounts of VLPs were biomineralized using the four methods under the same conditions and the biomineralized VLPs were centrifuged at 13,000 rpm× *g* for 10 min at 4 °C. VLPs in the supernatant and the mineralization efficiency were calculated. The sizes and zeta potentials of the VLPs-CaP complexes were determined with TEM and DLS (Table 1).

**Figure 1:**
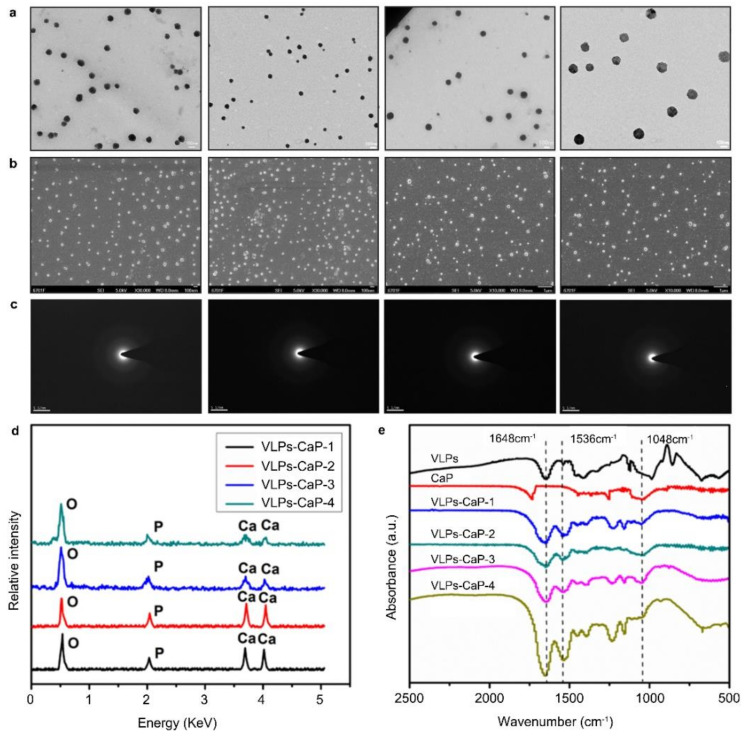
Characterization of the VLPs-CaP complexes. (**a**) TEM images demonstrating homogeneity of the VLPs-CaP complexes after biomineralization. (**b**) TEM images of the VLPs-CaP complexes. (**c**) Selected area electron diffraction images of the VLPs-CaP complexes demonstrating that the biomineralized shells were amorphous. (**d**) EDX analyses of the VLPs-CaP complexes demonstrating that the biomineralized shells were rich in Ca, P, and O. (**e**) FTIR spectroscopy showed that the VLPs-CaP complexes contained amorphous CaP.

The TEM SEM (Figure 1a,b, respectively) revealed that the four VLPs-CaP complexes were all uniformly dispersed with particle sizes of about 72.81–86.60 nm.

Selected area electron diffraction analysis showed that the biomineral shells of the four VLPs-CaP composites were amorphous (Figure 1c). Energy dispersive spectroscopy showed that the biomineralized shells were rich in Ca, P, and O (Figure 1d). FTIR spectroscopy confirmed that the four VLPs-CaP complexes contained both a phosphate moiety (from CaP) and an amino acid group (from VLPs) compared to CaP and VLPs alone (Figure 1e).

Biomineralization must ensure the availability of VLPs, so the effects of biomineralization on the biological characteristics of VLPs were analyzed. The results of ultraviolet-visible absorption spectrum and electronic circular dichroism indicated that biomineralization had no effect on the primary and secondary structures of the VLPs (Supplementary Figure S2). These results confirmed that all four biomineralization approaches successfully generated amorphous CaP shells on the surfaces of the VLPs.

### 3.2. Biocompatibility of VLPs-CaP Complexes

**Figure 2:**
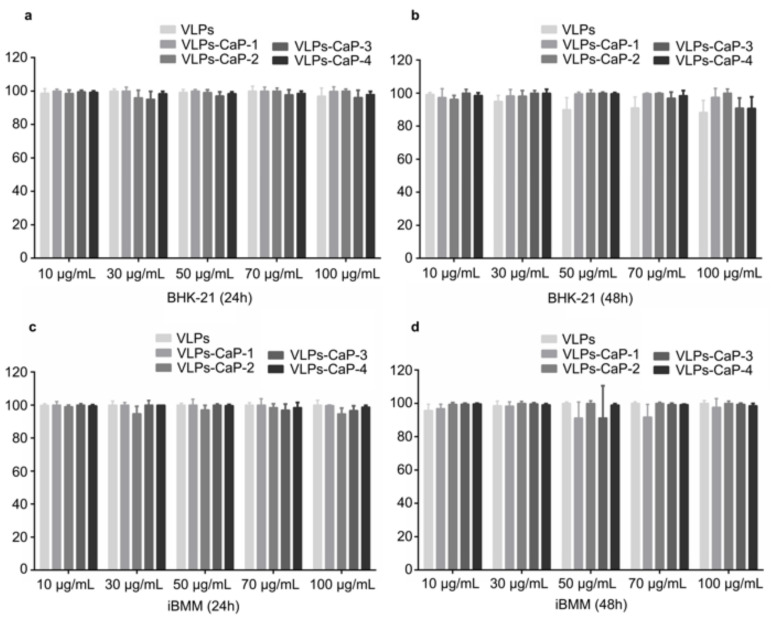
Cytotoxicity of the VLPs-CaP complexes. BHK-21 cells were treated with the VLPs-CaP complexes at concentrations of 10–100 µg/mL for 24 h (**a**) and 48 h (**b**). iBMM cells were treated with the VLPs-CaP complexes at concentrations of 10–100 µg/mL for 24 h (**c**) and 48 h (**d**). Multiple comparisons were performed using one-way ANOVA, followed by Bonferroni’s post-test, and *p* values <0.05 were considered significant.

Acceptable biocompatibility is a crucial feature of biomaterials and excellent biocompatibility is essential for vaccine preparations, thus the cytocompatibility was assessed to evaluate the biocompatibility of the designed VLPs-CaP. CaP, which is the main component of human bones and teeth, has excellent biocompatibility. In addition, the cytotoxicity of the four VLPs-CaP complexes was analyzed. The viability of BHK-21 cells and iBMM cells in response to various concentrations of VLPs was evaluated using a standard MTS assay, which showed that more than 85% of the cells were viable for all particle concentrations after incubation for 24 h and 48 h, even at a concentration as high as 100 μg/mL (Figure 2). These results indicate that the four VLPs-CaP complexes were not significantly toxic to either of the cell lines.

### 3.3. Cellular Uptake of VLPs-CaP

**Figure 3:**
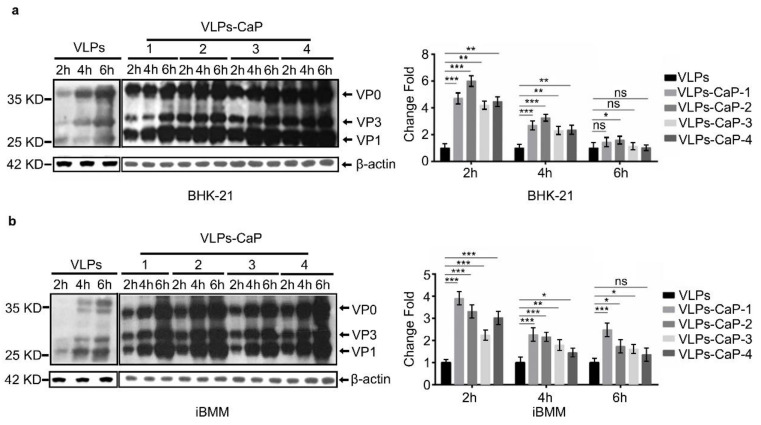
Cellular uptake of the VLPs-CaP complexes as quantified by western blot analysis. The BHK-21 cells (**a**) and iBMM cells (**b**) were treated with 5 µg of different VLPs for 2, 4, and 6 h to analyze the effect of biomineralization on cellular uptake. Multiple comparisons were performed using one-way ANOVA, followed by Bonferroni′s post-test, and *p* values < 0.05 were considered. *, *p* < 0.05; *, *p* < 0.01; ***, *p* < 0.001.

We aimed at examining whether the biomineralized VLPs could enter the cells as well as the intact one. The results showed that the four VLPs-CaP were effectively taken-up by both cell types. The western blot (Figure 3) showed that both VLPs-CaP and VLPs had entered the cells more easily with time and that the cells had taken-up the VLPs-CaP more readily than the VLPs, indicating that biomineralization promoted the entry of VLPs into the cell. The particle sizes of the four VLPs-CaP complexes were all around 81–86.60 nm and the zeta potentials were increased compared to the VLPs, which is conducive to interactions with negatively charged cell membranes.

### 3.4. Thermal Stability of VLPs-CaP Complexes

**Figure 4:**
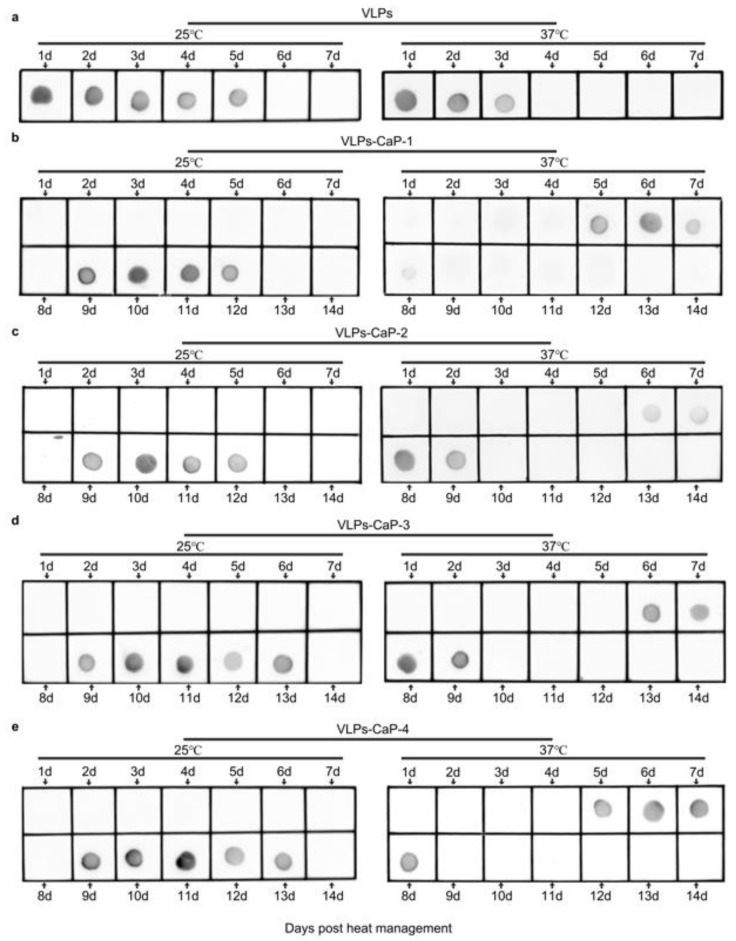
Thermo-resistance of the VLPs-CaP complexes. At 25 °C and 37 °C, VLPs were left for 1–7 days and VLPs-CaP for 1–14 days. VLPs in the supernatants of (**a**) VLPs, (**b**) VLPs-CaP-1, (**c**) VLPs-CaP-2, (**d**) VLPs-CaP-3, and (**e**) VLPs-CaP-4 were detected by dot blot analysis.

The effects of the biomineral shells on the thermal stability of the VLPs-CaP were investigated. The VLPs and the four VLPs-CaP complexes were stored at 25 °C and 37 °C for different periods of time, and the protein concentrations were determined by the dot blot analysis. As shown in Figure 4, the mineralized VLPs existed for at least four days at 37 °C, while the VLPs-CaP-2 and VLPs-CaP-3 existed for five days. There was no obvious difference among the four VLPs-CaP complexes at 25 °C for at least eight days.

**Figure 5:**
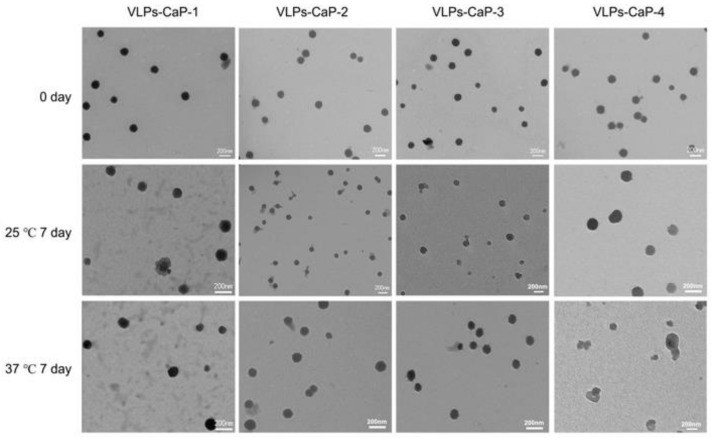
Long-term stability of the VLPs-CaP complexes. TEM images of CaP-VLPs-1, CaP-VLPs-2, CaP-VLPs-3, and CaP-VLPs-4 stored at different temperatures for different periods of time.

The morphologies of the four VLPs-CaP at 25 °C and 37 °C for seven days were observed using a TEM. As shown in Figure 5, the content of VLPs-CaP at 25 °C was higher than at 37 °C, and the contents of VLPs-CaP-2 and VLPs-CaP-3 were greater than those of VLPs-CaP-1 and VLPs-CaP-4. Further experimentation revealed that the VLPs-CaP were heat resistant (Figure S3).

### 3.5. Animal Experiments

The purpose of the animal experiments was to determine whether the mineralized VLPs could produce an immune response and whether the heat-treated VLPs-CaP could improve the immune response. Two animal experiments were conducted to evaluate the influence of biomineralization on the immune effects and the potential of biomineralization to generate thermostable vaccines.

**Figure 6:**
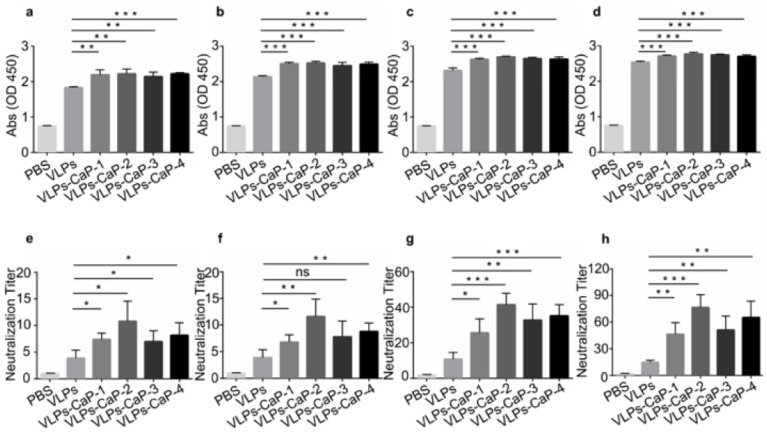
FMDV-specific immune responses and protection in guinea pigs. Groups of guinea pigs immunized with PBS, VLPs, CaP-VLPs-1, CaP-VLPs-2, CaP-VLPs-3, and CaP-VLPs-4. Blood samples were collected from the hearts of the guinea pigs on post-immunization days 7 (**a**), 14 (**b**), 21 (**c**), and 28 (**d**) for the detection of virus-specific Abs level. The titers of the neutralizing Abs were also measured on post-immunization days 7 (**e**), 14 (**f**), 21 (**g**), and 28 (**h**). Multiple comparisons were performed using one-way ANOVA, followed by Bonferroni’s post-test, and *p* values < 0.05 were considered significant. *, *p* < 0.05; **, *p* < 0.01; ***, *p* < 0.001.

To evaluate the influence of biomineralization on the immune effect, guinea pigs were immunized with PBS, VLPs, VLPs-CaP-1, VLPs-CaP-2, VLPs-CaP-3, and VLPs-CaP-4. Blood samples were collected from the hearts of the guinea pigs on post-immunization days 7, 14, 21, and 28. On post immunization 28, FMDV challenge was performed and the challenge protection rate was calculated. As shown in Figure 6a–d, the titers of specific Abs of each test group continuously increased with time with the exception of the control (PBS) group. Compared with the VLPs group, the titers of specific Abs were significantly increased in the VLPs-CaP-1, VLPs-CaP-2, VLPs-CaP-3, and VLPs-CaP-4 groups. The virus neutralization test was conducted to detect the titers of neutralizing Abs in guinea pig serum. The titers of neutralizing Abs showed a similar trend with the specific Abs among the groups (Figure 6e–h). The titers of the neutralizing Abs provided ideal protection after FMDV challenge. As shown in Table 2, the protection rate of all VLPs-CaP groups was ≥50%.

The protection rate of the VLPs, VLPs-CaP-2, and VLPs-CaP-3 groups was 75%. Moreover, the titers of neutralizing Abs were greater in the VLPs-CaP-1 and VLPs-CaP-4 groups than the VLPs group, but the challenge protection rate was lower than the differences among the individual groups. Therefore, a heat treatment experiment was conducted with an increased number of guinea pigs inoculated with the virus to improve the reliability of the results.

**Figure 7:**
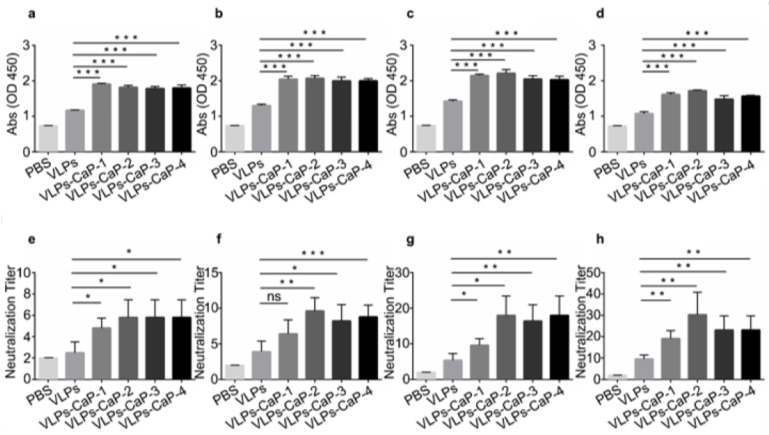
FMDV-specific immune responses and protection in guinea pigs. Groups of guinea pigs immunized with PBS, VLPs (37 °C for 2 days), CaP-VLPs-1 (37 °C for 4 days), CaP-VLPs-2 (37 °C for 4 days), CaP-VLPs-3 (37 °C for 4 days), and CaP-VLPs-4 (37 °C for 4 days). Blood samples were collected from the hearts of the guinea pigs on post-immunization days 7 (**a**), 14 (**b**), 21 (**c**) and 28 (**d**) to detect virus-specific Abs. The titers of the neutralizing Abs Were also measured on post-immunization days 7 (**e**), 14 (**f**), 21 (**g**) and 28 (**h**). Multiple comparisons were performed using one-way ANOVA, followed by Bonferroni´s post-test, and *p* values < 0.05 were considered significant. *, *p* < 0.05; **, *p* < 0.01; ***, *p* < 0.001.

To verify the heat-stability of the vaccines, guinea pigs were treated with PBS (as a control), heat-treated VLPs for two days, and heat-treated VLPs-CaP-1, VLPs-CaP-2, VLPs-CaP-3, and VLPs-CaP-4 for four days. The results showed that compared with the VLPs stored at 37 °C for two days, the four VLPs-CaP complexes stored at 37 °C for four days stimulated significantly higher titers of specific and neutralizing Abs in guinea pigs (Figure 7). On post-immunization days 21 and 28, the titers of specific Abs and neutralizing Abs were significantly increased in the biomineralized groups. The FMDV challenge results showed that the protection rate of the VLPs group was 16.7%, and the protection rate was higher in the four VLPs-CaP complex groups than that of the VLPs group (Table 3). These results indicate that biomineralization can produce a heat-stable vaccine against FMD.

## 4. Discussion

In this study, CaP biomineralized shells were artificially fabricated to improve the thermostability and immunogenicity of VLPs. The FMDV capsid is composed of 12 pentameric subunits, each of which is composed of five monomers and each monomer is composed of single copies of capsid proteins VP0 (composed of VP2 and VP4), VP1, and VP3. Analysis of the protein composition of the FMDV structural proteins with biological software showed that thee VP3, VP1, and VP0 proteins had 23, 18, and 27 highly acidic amino acid residues, respectively [30]. Negatively charged amino acids such as aspartic acid, glutamic acid, and phosphoserine are widely expressed in VLPs and play a critical role in controlling hydroxyapatite nucleation growth [31]. VLPs, as proteins, attract Ca^2+^ and ${\mathrm{PO}}_{4}^{3-}$ ions through their charged amino acid domains, and increase local supersaturation to a level sufficient to form a critical-sized nucleus, which can develop into hydroxy CaP crystals [32,33,34]. Inspired by biological mineralization in nature, the concentration and composition of acidic amino acids of natural FMD-VLPs were adjusted in the biomineralization solution in order to successfully prepare CaP biomineralization shells on the VLPs under mild physiological conditions.

The results of TEM, EDX, and FTIR analyses showed that the CaP biomineralized shells were successfully added to the surfaces of the VLPs. The results of DLS, TEM, and zeta potential analyses showed that the particle sizes and zeta potentials of the biomineralized VLPs were increased compared to those of the native VLPs. CaP is a highly biocompatible, biodegradable, and safe material with comparatively low toxicity and, thus is appropriate for use in human vaccines [35,36]. The experimental results also showed that VLPs-CaP-1, VLPs-CaP-2, VLPs-CaP-3, and VLPs-CaP-4 conveyed no obvious cytotoxicity. Compared with VLPs, VLPs-CaP can enter cells more easily, possibly because the zeta potential of biomineralized VLPs is higher than that of natural VLPs, which promotes interactions with the negatively charged cell membrane. In addition, the spontaneous release of closed VLPs from the CaP biomineralized shell is essential to ensure the availability of biomineralized vaccines. The CaP biomineralized shell has the advantages of protection and release due to its pH-dependent stability. A previous study reported that CaP is stable at physiological pH [37]. When the biomineralized vaccine is absorbed and presented by the cell, the CaP biomineralized shell usually degrades spontaneously upon contact with the acidic contents of endosomes and lysosomes [38]. The results of the present study show that the biomineralized VLPs were more readily taken-up by cells. Previous experiments have shown that the CaP biomineralized shell can be cleaved in a low pH environment, suggesting that VLPs-CaP can better activate the immune system to produce more Abs.

The FTIR spectroscopy and selected area electron diffraction results showed that the CaP biomineralized shells of biomineralized VLPs were amorphous. Amorphous biominerals are widely present in various organisms to improve tolerance to high temperature and salt stress [39,40]. Studies have shown that amorphous silica coatings and amorphous CaP coatings enhance the thermal stability of the vaccines [37,41]. The results of the present study showed that the thermal stability of biomineralized VLPs was significantly greater than that of native VLPs. The dot blot assay showed that heat-treated biomineralized VLPs was detectable for at least the first four days, while VLPs was only detectable for the first two days and then undetectable by Abs due to antigen destruction. Therefore, biomineralized VLPs stored at 37 °C for four days can stimulate the body to produce more protective Abs than native VLPs stored at 37 °C for two days. Fungi, archaea, and some plants can resist extreme cold, heat, and other factors by reducing the water contents [42]. The CaP biomineralized shells largely prevented the direct contact of the core of VLPs with the environment, thereby reducing the impact of external thermal motion. CaP biomineralized shells provide a physical barrier for the core of VLPs to prevent structural changes. The polyvalent calcium ions and phosphate ions of the CaP biomineralized shell can also induce electrostatic adsorption among the polar amino acids of VLPs, thereby enhancing the stability of VLPs in extreme environments. These mechanisms may explain the improved heat resistance of biomineralized VLPs.

An essential requirement of protein vaccines is the effective delivery of protein antigens to dendritic cells and to activate the adaptive immune response. Previous studies have reported that nanoparticles smaller than 100 nm in diameter preferentially drain to the lymph nodes upon encountering a high concentration of immature dendritic cells. The results of DLS, TEM, and SEM showed that the particle sizes of the four biomineralized VLPs were all around 80–100 nm, and thus can be adequately captured by dendritic cells. Animal experiments to assess the immune effect of biomineralized VLPs showed that biomineralized VLPs can induce more durable specific immune responses against FMDV than native VLPs. Since challenge experiments are relatively expensive, only four guinea pigs were randomly selected from each test group. Nonetheless, the challenge rate of the VLPs-CaP-1 and VLPs-CaP-4 groups was lower than that of the native VLPs group, although the challenge protection rate of all test groups was greater than 50%. Further animal experiments to assess the thermal stability of the biomineralized VLPs showed that storage at 37 °C for four days can induce more robust specific immune responses against FMDV than native VLPs stored at 37 °C for two days. Challenge experiments randomly selected from each experimental group showed that the challenge protection rate of all biomineralized VLPs groups was higher than that of the native VLPs group, indicating that biomineralization improved the immunogenicity of VLPs as an effective strategy for the production of thermostable VLPs vaccines.

The cost for the cold chain transport of vaccines is substantial. The results of this study showed that the CaP biomineralization improved the heat resistance of VLPs and produced more Abs. These excellent effects are very important to maintain the heat stability of vaccines and reduce dependence on a cold chain system, which will greatly reduce overall costs. Due to the lack of nucleic acids, VLPs are safer than viral vaccines. These characteristics are especially beneficial to the elimination of diseases. On 25 September 2020, a portion of the influenza vaccine supply of South Korea was exposed to room temperature during the cold chain transportation process, which caused the government to halt the pandemic influenza vaccination program, although an estimated 2296 people received the problematic influenza vaccination. The results of this study provide a feasible strategy to improve the heat resistance of vaccines and reduce the dependence on a cold chain system, which will be especially useful in developing countries with underdeveloped cold chain systems.

## Figures and Tables

**Table 1:** Basic characterization of the VLPs-CaP complexes.

Particles	Diameter [nm]	Zeta Potential [mV]	Biomineralization Efficiency (%)
VLPs	37.32	−20.9	
VLPs-CaP-1	72.81	−12.6	77.57%
VLPs-CaP-2	79.30	−13.0	75.42%
VLPs-CaP-3	86.60	−12.5	68.25%
VLPs-CaP-4	79.83	−13.0	71.82%

**Table 2:** Protection of guinea pigs after challenge with FMDV.

Vaccine	Number of Animals	Morbidity Number	Morbidity (%)	Protection Ratio (%)
PBS	4	4	100	0
VLPs	4	1	25	75
VLPs-CaP-1	4	2	50	50
VLPs-CaP-2	4	1	25	75
VLPs-CaP-3	4	1	25	75
VLPs-CaP-4	4	2	50	50

**Table 3:** Morbidity of guinea pigs immunized with different heat-treated vaccines.

Vaccine	Number of Animals	Morbidity Number	Morbidity (%)	Protection Ratio (%)
PBS	6	6	100	0
VLPs-37 °C-2 day	6	5	83.3	16.7
VLPs-CaP-1-37 °C-4 day	6	4	66.7	33.3
VLPs-CaP-2-37 °C-4 day	6	3	50	50
VLPs-CaP-3-37 °C-4 day	6	3	50	50
VLPs-CaP-4-37 °C-4 day	6	4	66.7	33.3

## Supplementary Materials

The following are available online at https://www.mdpi.com/article/10.3390/vaccines9080891/s1, Figure S1: Characterization of VLPs. (a) The particle size distribution of FMD VLPs. (b) TEM image of phosphotungstic acid negatively stained FMD VLPs. Figure S2: Characterization of VLPs and different VLPs-CaP. (a) Ultraviolet-visible (UV–Vis) absorption spectrum of VLPs, VLPs-CaP-1, VLPs-CaP-2, VLPs-CaP-3, and VLPs-CaP-4. (b) Electronic circular dichroism (ECD) of VLPs, VLPs-CaP-1, VLPs-CaP-2, VLPs-CaP-3, and VLPs-CaP-4. Figure S3: Characterization of VLPs, different VLPs-CaP, and different VLPs + CaP.
