# Supplementary material for: Four Simple Biomimetic Mineralization Methods to Improve the Thermostability and Immunogenicity of Virus-like Particles as a Vaccine against Foot-and-Mouth Disease
Source: Vaccines (Basel). 2021 Aug 12;9(8):891. doi: 10.3390/vaccines9080891 (PMC8402440; doi:10.3390/vaccines9080891)
Supplement: Supplementary file 1 [file vaccines-09-00891-s001.zip › vaccines-1282478-supplementary.pdf]

Four Simple Biomimetic Mineralization Methods to Improve the Thermostability and Immunogenicity of Virus-like Particles as a Vaccine against Foot-and-Mouth Disease

Mengnan Guo [1,†], Jiajun Li [1,†], Zhidong Teng [1], Mei Ren [1], Hu Dong [1], Yun Zhang [1], Jiaxi Ru [1], Ping Du [1], Shiqi Sun [1,2] and Huichen Guo [1,2,3,*]

[1] State Key Laboratory of Veterinary Etiological Biology, National Foot-and-Mouth Disease Reference Laboratory, Lanzhou Veterinary Research Institute, Chinese Academy of Agricultural Sciences, Lanzhou 730030, China; guomengnan2021@163.com (M.G.); jiajunlii@163.com (J.L.); tengzhidong163@163.com (Z.T.); Mei.Ren@student.uliege.be (M.R.); donghu.0608@163.com (H.D.); zhangyun03@caas.cn (Y.Z.); rujiaxi@caas.cn (J.R.); westernboydp555@163.com (P.D.); sunshiqi@caas.cn (S.S.)

[2] College of Animal Science, Yangtze University, Jingzhou 434025, China

[3] Yunnan Tropical and Subtropical Animal Virus Diseases Laboratory, Yunnan Animal Science and Veterinary Institute, Kunming 650000, China

* Correspondence: guohuichen@caas.cn

† These authors contributed equally to this work.

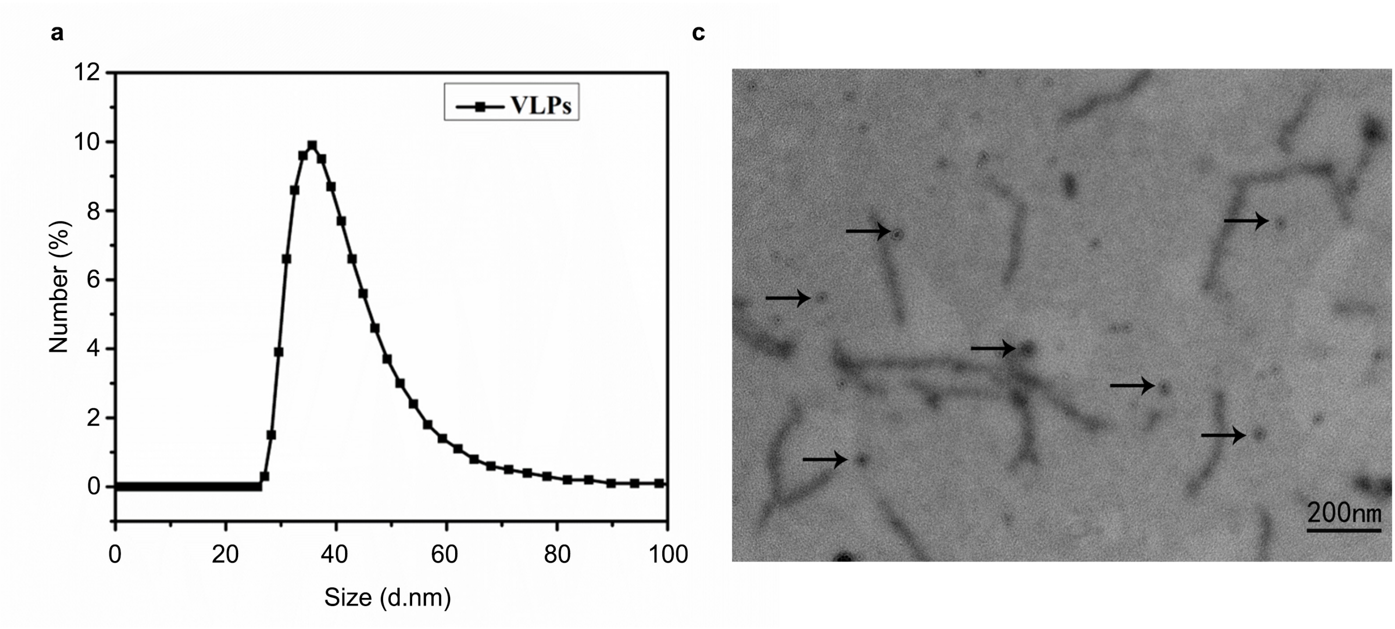

Figure S1. Characterization of VLPs. (**a**) The particle size distribution of FMD VLPs. (**b**) TEM image of phosphotungstic acid negatively stained FMD VLPs.

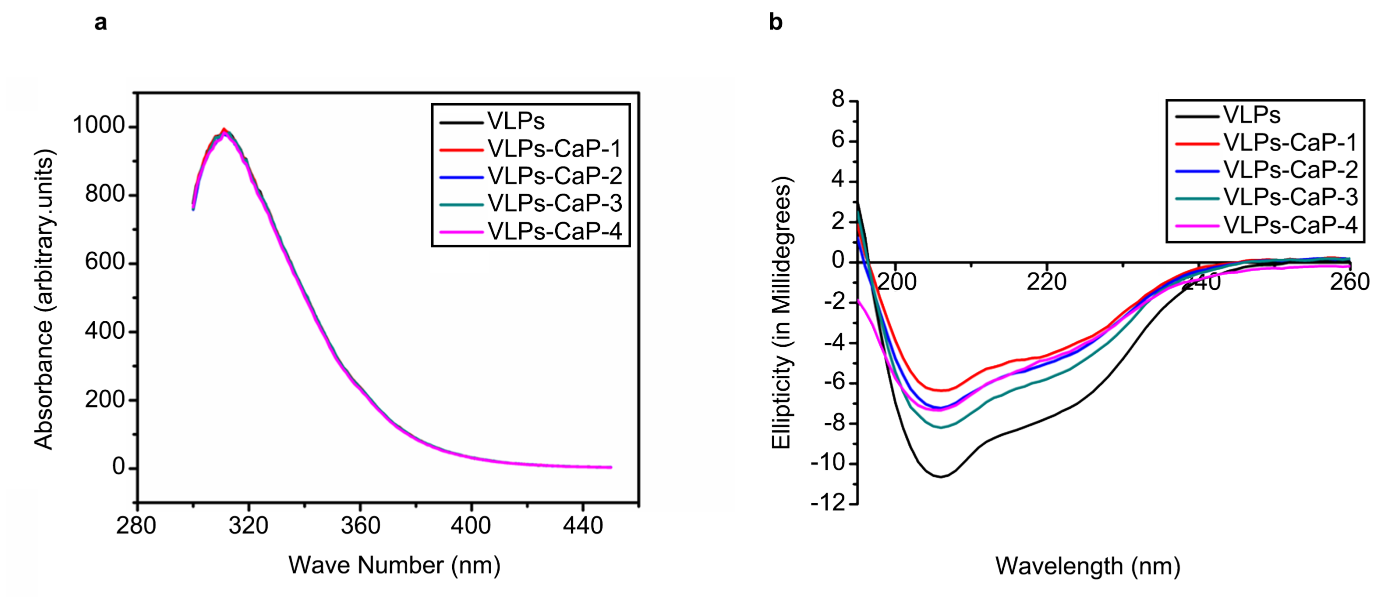

Figure S2. Characterization of VLPs and different VLPs-CaP. (**a**) Ultraviolet-visible (UV-vis) absorption spectrum of VLPs, VLPs-CaP-1, VLPs-CaP-2, VLPs-CaP-3 and VLPs-CaP-4. (**b**) electronic circular dichroism (ECD) of VLPs, VLPs-CaP-1, VLPs-CaP-2, VLPs-CaP-3 and VLPs-CaP-4.

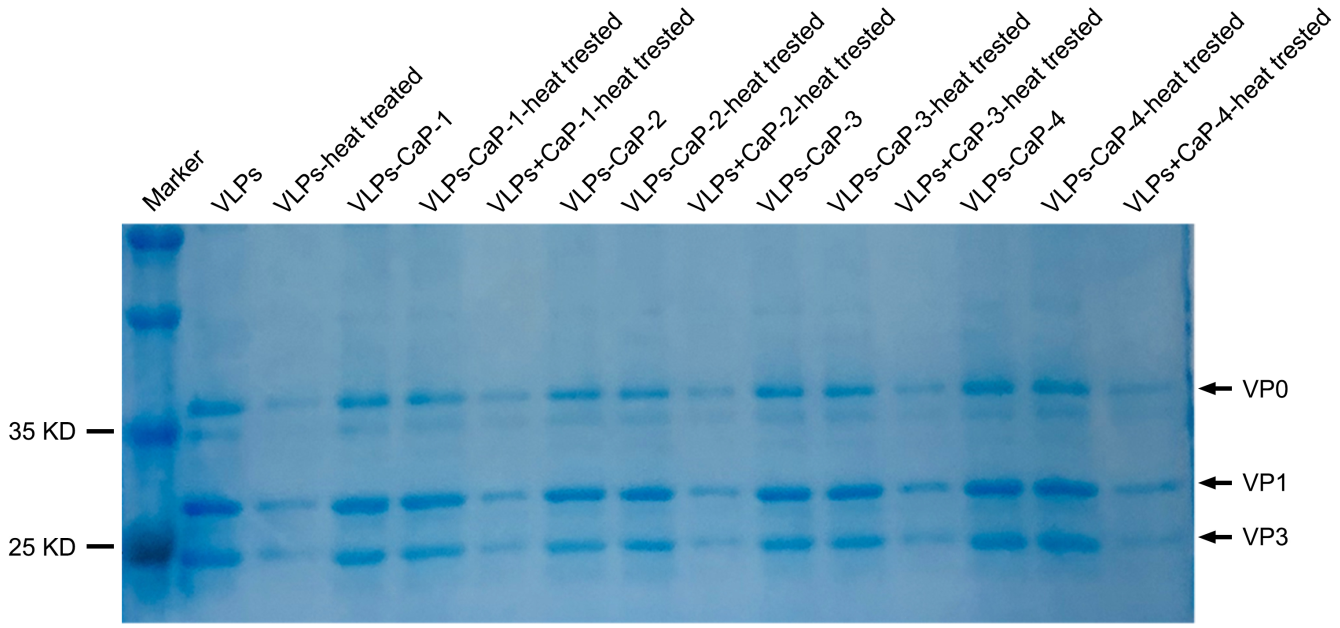

Figure S3. Characterization of VLPs, different VLPs-CaP and different VLPs+CaP.
